# Robust Pedestrian Tracking and Recognition from FLIR Video: A Unified Approach via Sparse Coding

**DOI:** 10.3390/s140611245

**Published:** 2014-06-24

**Authors:** Xin Li, Rui Guo, Chao Chen

**Affiliations:** 1 Lane Department of CSEE, Morgantown, WV 26506-6109, USA; 2 Department of EECS, University of Tennessee, Knoxville, TN 37996, USA; E-Mail: rguo1@utk.edu; 3 Department of Electrical and Computer Engineering, University of Missouri, Columbia, MO 65211, USA; E-Mail: ccwwf@mail.missouri.edu

**Keywords:** robust tracking, pedestrian recognition, sparse coding, template updating, FLIR video

## Abstract

Sparse coding is an emerging method that has been successfully applied to both robust object tracking and recognition in the vision literature. In this paper, we propose to explore a sparse coding-based approach toward joint object tracking-and-recognition and explore its potential in the analysis of forward-looking infrared (FLIR) video to support nighttime machine vision systems. A key technical contribution of this work is to unify existing sparse coding-based approaches toward tracking and recognition under the same framework, so that they can benefit from each other in a closed-loop. On the one hand, tracking the same object through temporal frames allows us to achieve improved recognition performance through dynamical updating of template/dictionary and combining multiple recognition results; on the other hand, the recognition of individual objects facilitates the tracking of multiple objects (*i.e.*, walking pedestrians), especially in the presence of occlusion within a crowded environment. We report experimental results on both the CASIAPedestrian Database and our own collected FLIR video database to demonstrate the effectiveness of the proposed joint tracking-and-recognition approach.

## 1. Introduction

The capability of recognizing a person at a distance in nighttime environments, which we call remote and night biometrics, has gained increasingly more attention in recent years. Fast advances in sensor technology (e.g., infrared cameras) and biometric systems (e.g., video-based recognition) have facilitated the task of remote and night biometrics. Object tracking and recognition are two basic building blocks in almost all video-based biometrics systems, including forward-looking infrared (FLIR)-based ones. The literature of object detection/tracking, face recognition and visual surveillance is huge; for recent advances, please refer to [1–3] and their references; pedestrian detection and tracking from FLIR video has also been studied in [4–7]. However, the relationship between detection/tracking and recognition has not been well studied in the literature. To the best of our knowledge, joint tracking and recognition has been considered under the context of particle filtering [8] only and specifically in the scenario of face biometrics [9].

In this paper, we propose to tackle joint object tracking and recognition under a unified sparse coding-based framework. Sparse coding originated from the research on compressed sensing theory [10] and has been recently leveraged into the problems of robust object tracking [11–13] and robust face recognition [14, 15]. For both tracking and recognition problems, the target patch/template of interest is sparsely represented in the space spanned by the dictionary (a collection of matching templates); and the final result is given by the candidate with the smallest projection error. Such a similarity motivates us to cast the two problems under the same framework and solve them simultaneously, *i.e.*, unlike previous works assuming a dictionary of templates (e.g., face portions) already cropped from the original image/video, ours obtains this dictionary by dynamically tracking the target of interest (e.g., a walking pedestrian).

We argue that tracking and recognition can benefit from each other for the following reasons. On the one hand, robust tracking of an object under a particle filter framework [16] often involves the updating of the matching templates on-the-fly. Such a dynamical strategy of template updating helps overcome the difficulties with occlusion and the cluttered background, which are also common adversary factors to the task of robust recognition. Moreover, persistently tracking allows the system to temporally combine the recognition results across multiple frames for improved accuracy (since we know it is the same object that has been tracked) [17, 18]. On the other hand, high-level vision tasks, such as recognition, often facilitates those at lower levels, including tracking, especially in the situation of multiple targets being involved [19]. More specifically, we suggest that the recognition result can be exploited by the template updating strategy to better fight against occlusion and a cluttered background. Such tracking-by-recognition offers some new insight to the challenging problem of multi-target tracking, which was often tackled by an energy optimization approach [20].

When applied to remote and night biometrics systems, the proposed approach has several advantages over other competing ones (e.g., gait-based [21] or silhouette-based [22]). First, previous approaches mostly count on image/video segmentation to extract relevant gait or silhouette information before recognition; consequently, segmentation errors have a significant impact on the accuracy of recognition [23]. By contrast, the proposed one directly works with image patches and does not involve any cropping or segmentation at all (note that in many previous works, such as [14], it is assumed that cropped image patches are already available). Second, it is widely known that occlusions and background clutters are often primary obstacles to various vision tasks, including tracking and recognition. Sparse coding has shown great potential in fighting against those adversary factors, thanks to the power of collaborative representation [15] (please refer to the Experimental Results section). Third, the unification of tracking and recognition allows us to jointly optimize these intrinsically connected components, which is highly desirable in the scenario of handling complicated cases, such as multi-target tracking in a crowd [24]. In other words, tracking and recognition can be viewed as two sides of the same coin: One helps the other and *vice versa*.

## 2. Background on Sparse Coding

In this section, we review the current state-of-the-art in sparse coding and its applications into object tracking/recognition [25]. The basic idea behind sparse coding is to approximate a signal of interest **x** ∈ *R^n^* by linear combination of a small number of atoms (elements in a dictionary **A**_*m*×*n*_); namely, **x**_*m*×1_ = **D**_*m*×*n*_**a**_*n*×1_, where **a** is the vector of sparse coefficients. Ideally, the sparsity constraint is enforced about the total number of nonzero coefficients in **a**, which gives rise to the following constrained optimization problem:

(1) $$\underset{\text{a}}{\min}{\left\|\text{a}\right\|}_{0}\text{subject}\;\text{to}\left\|\text{x}-\text{Aa}\right\|\leq \in$$

However, the above problem is known to be NP-hard [26], and it is often suggested that the original *l*_0_-norm be replaced by its *l*_1_ counterpart. That is, one considers the following computationally tractable formulation:

(2) $$\underset{\text{a}}{\min}{\left\|\text{a}\right\|}_{1}+\left\|\text{x}-\text{Aa}\right\|$$

where λ is the Lagrangian multiplier converting the constrained optimization into an unconstrained one [27]. Various algorithms have been developed in recent years to solve this class of *l*_1_-minimization problems (for a recent review, please refer to [28] and its references). Meanwhile, it is amazing to witness that many engineering problems across different disciplines can be reformulated into a variant of *l*_1_-minimization problem. Within the scope of this paper, we opt to review two of them; namely, object tracking and object recognition.

### 2.1. Sparse Coding for Object Tracking

**Figure 1.**
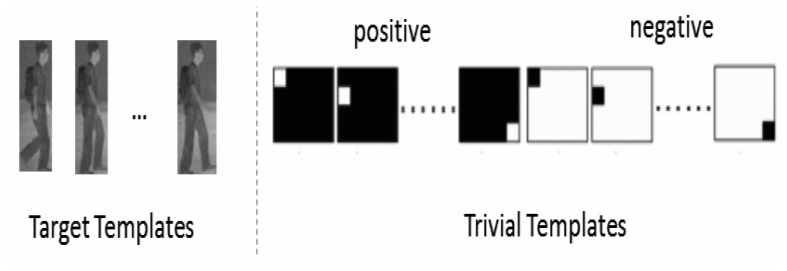
Decomposition of a dictionary into target and trivial templates in sparse coding-based object tracking.

The fundamental assumption for appearance-based object tracking is that the global appearance of an object, despite varying illumination and viewpoint conditions, is still characterized by a low-dimensional space. Under the context of appearance-based object tracking, dictionary **A** is decomposed of target templates (image patches in *R^m^*), as well as a collection of trivial templates (to model occlusion and noise in the real-world observation data), as shown in Figure 1. If one writes **A** as:

(3) $${\text{x}}_{m\times 1}=[\text{T}\;\text{I}-\text{I}]{\left[\text{b}\;{\text{e}}^{+}{\text{e}}^{-}\right]}^{t}={\text{A}}_{m\times (n+2m)}{\text{a}}_{(n+2m)\times 1}$$

where **T** = [**t**_1_, …, **t***_n_*] denotes *n* target templates (note that *m* >> *n*) and **e**^+^, **e**^−^ ∈ *R^m^* correspond to positive/negative trivial coefficient vectors, respectively.

For a good target candidate, there are only a small number of nonzero coefficients in positive and negative trivial coefficients accounting for the noise and partial occlusion. Such an observation has led to the formulation of object tracking into a *l*_1_-minimization problem, as proposed in [11, 29-31]. The final tracking result is obtained by finding the smallest residual after projecting onto the subspace spanned by target templates, *i.e.*, ‖**x** − **Tb**‖_2_. Under a particle filtering framework [16], such minimum-error tracking admits a maximum *a posteriori*probability interpretation. Further improvement on robustness tracking can be brought by the idea of template updating. More specifically, the *l*_2_-norm of template **t**_i_ intuitively indicates its significance to tracking; therefore, it is a plausible to eliminate the template of the least weight and replace it by the newly-obtained successful tracking result.

### 2.2. Sparse Coding for Object Recognition

Based on a similar observation to tracking, one can assume that the appearance of each individual subject lies in a unique low-dimensional subspace, and the structure of this subspace can be exploited to distinguish the subject of interest from others [14]. Therefore, if we consider a collection of *k* subjects, each containing *n* templates **t**_*i, j*_ ∈ *R^m^* (again, *m* is the size of the template of the image patch), the dictionary **A**_*m*×*N*_ will consist of *N* = *nk* elements. For any given inquiry template **x**, one can formulate the following sparse coding problem:

(4) $$\underset{\text{a}}{\min}{\left\|\text{a}\right\|}_{1}+\lambda \left\|{\text{x}}_{m\times 1}-{\text{A}}_{m\times N}{\text{a}}_{N\times 1}\right\|$$

where sparse coefficients a will be exploited to tell which subspace the inquiry is associated with. Ideally, the sparsest solution will associate the inquiry with the group of templates from a single subject class. However, due to noise and modeling errors, inference from other competing classes might arise; in other words, one might observe small nonzero entries associated with several subject classes. Therefore, it is often desirable to identify the subject by a twist of the above minimum-error strategy; namely, one can calculate the residual errors after projecting onto the subspace spanned by each class of target templates [14]:

(5) $$E(i)=\left\|{\text{x}}_{m\times 1}-{\text{A}}_{m\times N}\delta_{\left(i\right)}({\text{a}}_{N\times 1})\right\|$$

where *δ*_(*i*)_ (**a**) is the characteristic function that assigns ones to the entries associated with subject *i* in **a**. Then, the identity of inquiry **x** is obtained by *Id* = argmin*_i_ E*(*i*), 1 ≤ *i* ≤ *k*.

As articulated in [15], it is the idea of collaborative representation—namely, the formulation of joint dictionary A—that contributes to the good performance of Equation (5) in robust face recognition. It has been shown that replacing *l*_1_-norm by its *l*_2_-counterpart achieves comparable recognition performance, even though the computational complexity of the solution algorithm can be dramatically reduced (since the regularized least-square problem admits the analytical solution). When compared against previous *l*_2_-based approaches (e.g., eigen-face [32]), we note that it is collaborative representation that enforces the global constraint on the collection of appearance subspaces spanned by individual subjects. In other words, the competition among sparse coefficients *a_i_* contributes to the effectiveness of the winner-take-all strategy, and therefore, it is possible to obtain robust recognition by searching for the smallest projection errors.

Despite the use of sparse coding in both object tracking and recognition, it should be emphasized that the relationship between them has not been studied in the open literature. To the best of our knowledge, joint tracking-and-recognition has only been addressed in two isolated scenarios: one is to embed them into a single particle filtering framework [8], and the other is to integrate tracking with recognition specially for the class of face biometrics [9]. The apparent similarity between Equations (3) and (5) inspires us to explore a unified sparse coding-based approach toward joint tracking-and-recognition. The primary objective of this paper is to demonstrate that such a joint approach can offer several new insights into the design of robust vision systems and find niche applications in challenging environments, such as remote and night biometrics using FLIR data.

## 3. Joint Tracking-and-Recognition: A Unified Approach via Sparse Coding

In this paper, we formally define a joint tracking-and-recognition problem as follows. Given an inquiry FLIR video *X* containing walking pedestrians and a database of *k* subjects each associated with *n* video segments (training samples), establish the identity of the inquiry video. Note that unlike previous studies, [8] and [9], in which only one subject is considered, tracking and recognition are more tightly twisted in our multi-subject formulation (*i.e.*, one has to simultaneously track and recognize multiple subjects). At first sight, the interference among multiple subjects (e.g., one person could become occluded due to another person's presence) makes the joint tracking-and-recognition problem a lot more challenging than the single-subject scenario. To overcome this difficulty, we propose to gain a deeper understanding between tracking and recognition in this section.

### 3.1. Tracking-for-Recognition: Exploiting Temporal Redundancy

We first consider a simplified scenario where only one walking pedestrian is present in the inquiry video. When no interference is present, tracking a single pedestrian is a solved problem, and the recognition subproblem can be solved by sparse coding in a similar fashion to face recognition [14]. A more interesting question is: how can tracking help recognition? Here, we present a Bayesian interpretation of sparse coding-based recognition [14], which facilitates the exploitation of temporal redundancy arising from tracking a target template in the inquiry video. The key observation behind tracking-for-recognition lies in the fact that if it is known as *a priori* that multiple templates are associated with the same identity, such information can be exploited by the recognition system to improve the accuracy Each template can be viewed as an independent classifier, and accordingly, the idea of combining classifiers [33] can be easily implemented under the sparse coding framework.

Following the same notation used above, we consider a dictionary **A**_*m*×*N*_ consisting of *k* subjects each containing *n* templates **t**_*i*, *j*_ ∈ *R^m^* (*N* = *nk*). The subspace constraint of the appearance model for subject *i* (1 ≤ *i* ≤ *k*) implies that a target template **x** associated with subject *i* can be best approximated by the following sparse coding strategy:

(6) $$\text{x}\approx \text{A}\delta_{\left(i\right)}(\text{a}),$$

where *δ*_(*i*)_ (**a**) is a binary vector in *R^N^*, whose only nonzero elements are located at *j* = (*i* — 1) * *n* + 1, …, *i* * *n* (*i.e.*, those associated with subject *i*). If the approximation error is given by *E*(*i*) = **x** — **A**_*m*×N_ δ_(*i*)_ (**a**_*N*×1_) and assumed to observe an i.i.d. Gaussian model $N(0,\sigma_{w}^{2})$, then the likelihood function of observing a template **x***_i_* given subject *i* (denoted by *w_i_*) can be written as:

(7) $$p(\text{x}|w_{i})\approx \text{exp}\left(-\frac{{\left\|E(i)\right\|}_{2}^{2}}{2\sigma_{w}^{2}}\right)$$

Now, it follows from the Bayesian formula that the maximum *a posteriori* (MAP) classification of a given template **x** can be obtained from:

(8) $$\underset{i}{\max}p(w_{i}|\text{x})=\underset{i}{\max}\frac{p(\text{x}|w_{i})p(w_{i})}{p(\text{x})}$$

which implies the equivalence between the MAP strategy in the Bayesian classifier and the minimum-distance classifier of Equation (5) used in SCR. Such a connection allows us to conveniently exploit the temporal redundancy of an inquiry video under the framework of combining classifiers, as we will elaborate next.

Similar to the setup in [33], we use {*w*_1_, …, *w_k_*} to denote *k* different classes of subjects/identities and {**x**_1_, …, **x***_l_*} the collection of measurement vectors. Given an inquiry FLIR video *X*, those measurement vectors are obtained by tracking a single target template **x** across multiple frames. Therefore, a Bayesian classifier works by assigning the label *Id =* max*_i_ p*(*w_i_*|**x**_1_, …, **x***_l_*), which, in turn, can be written as:

(9) $$\underset{i}{\max}p(w_{i}|{\text{x}}_{1},\;\ldots ,{\text{x}}_{l})=\underset{i}{\max}\frac{p({\text{x}}_{1},\;\ldots ,{\text{x}}_{l}|w_{i})p(w_{i})}{p({\text{x}}_{1},\;\ldots ,{\text{x}}_{l})}$$

Under the assumption that all measurement vectors are conditionally statistically independent, we have:

(10) $$p({\text{x}}_{1},\;\ldots ,{\text{x}}_{l}|w_{i})=\prod_{j=1}^{l}p({\text{x}}_{j}|w_{i})$$

Substituting Equations (7) and (8) into Equation (10), we can obtain the so-called feature-level fusion strategy:

(11) $$p({\text{x}}_{1},\ldots ,{\text{x}}_{l}|w_{i})\approx \text{exp}\left(-\frac{{\sum_{j=1}^{l}\left\|E_{j}(i)\right\|}_{2}^{2}}{2\sigma_{j}^{2}}\right)$$

Therefore, the MAP decision boils down to a generalized minimum-distance classifier defined with respect to the group of measurement vectors. Alternatively, as suggested in [33], one can combine the decision outcomes instead of posterior probabilities, e.g., the final decision can be made by either sum rule $\text{Id}={\mathrm{argmin}}_{i}{\sum_{j=1}^{l}\left\|E_{j}(i)\right\|}_{2}^{2}$, 1 ≤ *i* ≤ *k* or majority-vote rule *Id* = *mode* {*Id*_1_, …, *Id_l_*}, where *Id_j_* is the label returned by applying the minimum-distance classifier of Equation (8) to measurement vector **x_j_**. Even though the benefit of combining classifiers has been well-established in the literature (e.g., refer to [34]), the relationship between the number of classifiers *l* and performance gain is not. As we will show in the Experimental Results, even a small number of *l* (<10 frames) measurement vectors can dramatically boost the recognition accuracy.

### 3.2. Tracking-by-Recognition: Nonlocal Template Updating

Now, let us consider the more general situation: a multi-subject extension of the above joint tracking-and-recognition problem. In the literature, the problem of multi-object tracking is often addressed under the framework of energy minimization (e.g., refer to [35, 36] and their references). Two common technical challenges with tracking multiple objects is that the space of all possible trajectories is large and the appearance of a target might vary dramatically, due to the presence of occlusion or illumination variations. Consequently, it often requires special attention to design an appropriate cost function and a fast search strategy to solve the multi-object tracking problem. By contrast, we propose to cast multi-object tracking under the framework of sparse coding and explore the question of how the recognition result could help a multi-object tracking algorithm fight against adversary factors, such as occlusion and illumination variations. The basic assumption behind our tracking-by-recognition approach is that as long as the problem of multi-object tracking can be solved in a robust fashion, the recognition of multiple objects becomes straightforward (e.g., based on what we have discussed in the previous subsection on tracking-for-recognition).

The key observation behind our tracking-by-recognition is that one person's appearance along the moving trajectory behaves like the noise to the tracking of another person. For this reason, only the person of interest (that has been recognized) contributes to the formation of dictionary A in sparse coding-based tracking; all others can be handled the same way as background clutter. In other words, recognition facilitates the multi-object tracking problem by recognizing that for each appearance subspace of an individual subject, all other subjects, as well as the background can be modeled by the outliers. Such an observation leads us to rethink the template updating strategy proposed in [11], where the least-important template is eliminated from the dictionary and ω*_i_* = ‖**t***_i_*‖_2_ is adopted to quantify the importance of a template **t_i_**. Empirical studies have shown that such strategy is highly sensitive to occlusion, due to the reasons listed above. Instead, we propose a nonlocal alternative strategy of template updating; based on the recognition result, one can switch to a default set of templates upon the suspicion of occlusion. One way of implementing such a strategy is to save a copy of templates that have been recognized to be the same person (but likely in the distant history or even in the training set).

It is enlightening to appreciate the advantage of the above tracking-by-recognition formulation for multi-object tracking over existing energy minimization approaches. In energy minimization approaches, occlusion handling is often a thorny issue to address when coming up with an appropriate energy term for multi-object tracking. For example, a sophisticated global occlusion reasoning strategy is studied in [36], where a principled modeling of occlusion remains elusive, due to the complex dependency between a target's visibility and other targets' trajectories. By contrast, we argue that if the ultimate objective of the surveillance system is to recognize walking pedestrians, one can get around the tricky occlusion issue by stopping the tracker. In other words, the continuity of motion trajectory is unnecessary for the task of recognition; what matters is only the accumulated group size of measurement vectors (occlusion will reduce this size, but there is no need for accurate occlusion detection). In other words, tracking and recognition are essentially two sides of the same coin: tracking where a target template goes in the next frame is conceptually equivalent to recognizing whether a new hypothesized template in the next frame still belongs to the same class as the target one. With the recognition result available, tracking can always rely on a more trustworthy source (e.g., nonlocal rather than local) for template updating.

## 4. Experimental Results

### 4.1. Experimental Setup

In this section, we report our experimental results with two FLIR pedestrian databases: one is collected by CASIA (Dataset C in the CASIA Gait Database, Publicly available at http://www.cbsr.ia.ac.cn/english/Databases.asp), and the other is collected at the WVU Erickson Alumni Center (not publicly available, but it can be requested from http://www.citer.wvu.edu/biometric_dataset_collections). The CASIA Dataset C contains 153 subjects, each of which contains 11 video clips acquired by an FLIR camera. Each subject passes through the scene with and without carrying a bag, as well as at varying walking speeds; although silhouettes of those 153 subjects are supplied, we have found that they are error-prone, and therefore, we do not utilized them in our approach. The WVU dataset contains 30 subjects (18 males and 12 females) walking at three planned camera distances: 20, 25 and 30 m. In addition to the bag carrying option, the protocol includes both single-person and double-person scenarios. In the latter, two person walk toward each other, one carrying a bag and the other empty-handed; when they meet halfway, the bag will be handed to the other; then, they walk away from each other. Both occlusion and carrying a bag are adversary factors to pedestrian tracking and recognition in this setup.

To promote reproducible research, the source codes and saved experimental results accompanying this research can be accessed at http://www.csee.wvu.edu/ xinl/code/FLIR.zip. In our MATLAB-based implementation, we have built upon two previous releases of sparse coding for tracking and recognition. The source codes of sparse coding for *l*_1_-based tracking and recognition have been obtained from http://www.dabi.temple.edu/ hbling/code_data.htm#L1_Tracker and http://www.eecs.berkeley.edu/ yang/software/l1benchmark/. More specifically, the dictionary needed for sparse coding-based recognition is obtained from the tracking result; we simply normalize the cropped templates to a common size. For the CASIA Dataset C, the following parameter setting is adopted: *k* = 153, *n* = 40.

### 4.2. Single-Object and Multi-Object Tracking

**Figure 2.**
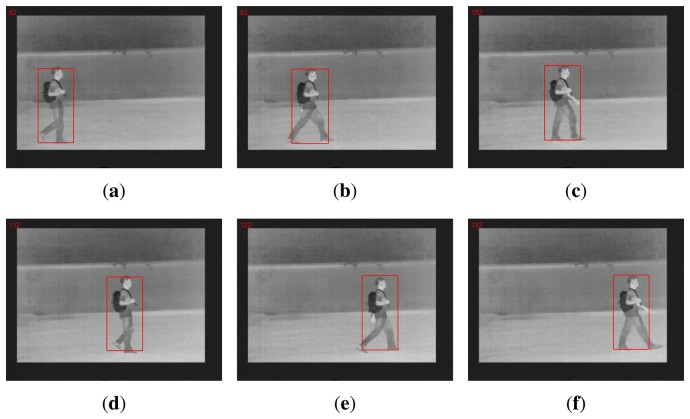
Sample tracking results for the forward-looking infrared (FLIR) video (red boxes highlight the locations of the walking pedestrian).

We first demonstrate the tracking result for single-object tracking. Figure 2 shows a collection of sample frames obtained from one typical FLIR video of CASIA Dataset C by *l*_1_-based tracking. Since the background is relatively simple and only one pedestrian is present, the tracking is not a challenging issue for this data set. The new insight supplied by this experiment lies in that *l*_1_-based tracking offers an automatic and robust cropping tool to obtain matching templates; *i.e.*, the elements of dictionary **A**. Note that the length of even a short video segment is a few seconds, which implies that at least dozens (or even hundreds) of matching templates can be cropped from the video clip. We note that this fact suggests that there is a significant amount of temporal redundancy that can be exploited by the recognition component.

**Figure 3.**
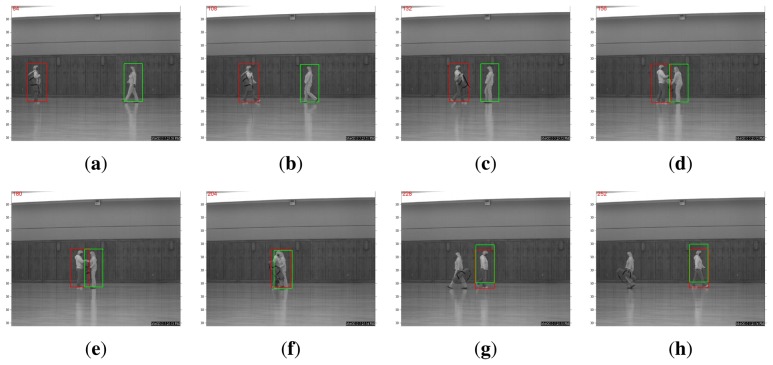
Tracking failure result obtained by [11] due to occlusion (after the two persons pass by each other, the tracking algorithm got confused; both red and green boxes get attached to the pedestrian walking to the right).

**Figure 4.**
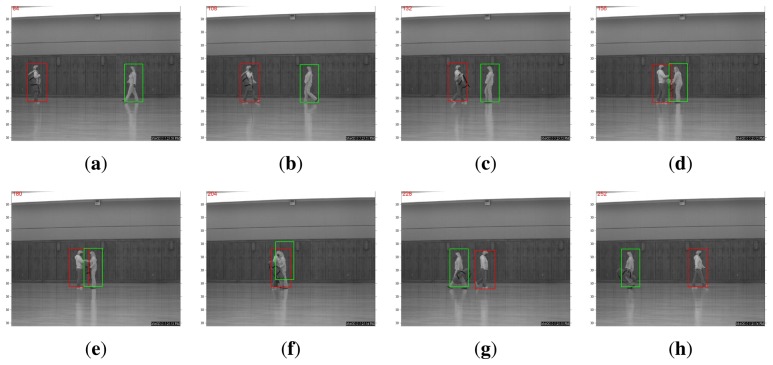
Joint tracking-and-recognition is capable of persistently tracking both pedestrians regardless of the occlusion and bad exchange (both red and green boxes are correctly associated with the correct identity).

A more interesting comparison result is in the scenario of multi-object tracking. For example, the WVU dataset contains test sequences in which two person walk toward each other. When the two pedestrians meet, one hands the bag to the other, and then, they continue walking away from each other. Such a protocol dictates that occlusion is present for a relatively long period of time. As shown in Figure 3, the straightforward application of the *l*_1_-based tracking algorithm in [11] expectedly fails at the occlusion. The algorithm will be confused by the overlap of target templates associated with two pedestrians. By contrast, a recognition-based, nonlocal, template-updating strategy proposed in the previous section can produce robust and accurate tracking, even after one person hands the bag to the other (note that there are significant variations in terms of appearance), as shown in Figure 4. This is because when occlusion occurs, the recognition-based strategy will update the template stored from a distance past (in other words, nonlocal becomes more trustworthy than the local temporal neighborhood). Such experimental results justify the effectiveness of our tracking-by-recognition approach.

### 4.3. Robust Pedestrian Recognition from FLIR Video

**Figure 5.**
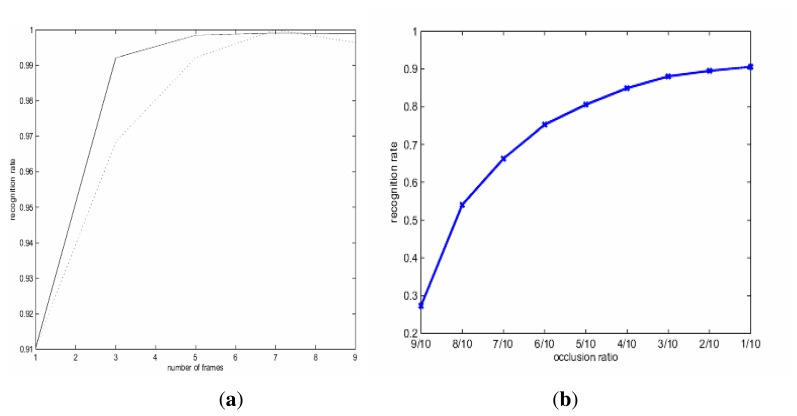
The recognition performance of sparse coding-based recognition: (**a**) exploiting temporal redundancy improves the recognition accuracy (solid: sum rule; dashed: majority voting); (**b**) the recognition performance gracefully degrades as the occlusion ratio increases (no temporal fusion involved *l* = 1).

Next, we report our experimental results with sparse coding-based recognition. In particular, we want to explore the gain brought by exploiting temporal redundancy (through combining classifiers) and the impact of occlusion on recognition performance. In the first experiment, we change the parameter *l*—the size of measurement vectors or the total number of frames for which we have successfully tracked for the inquiry video *X*. Two rules of combining the classification results have been implemented: sum *vs.* majority vote. Figure 5a shows how the accuracy of recognition evolves as l varies: it can be observed that the gain improves rapidly as l increases and quickly saturates. Therefore, even when a small number of measurement vectors (e.g., *l* = 9 or $<\frac{1}{3}$ second for 30 fps of video) is available, highly accurate recognition (close to 100%) is possible thanks to the power of temporal fusion. By contrast, we note that the best recognition performance reported for this data set is 96% in the open literature (e.g., gait energy image based [21]). Such a finding seems to suggest that video-based biometrics has a lot more potential than image-based, thanks to the blessing of redundancy.

In the second experiment, we artificially mask a certain percentage of the inquiry template (e.g., to simulate how the lower part of human body is occluded by bushes or deep grass in a real-world scenario) and test the performance of sparse coding-based recognition (no fusion is involved, *i.e.*, *l* = 1). Figure 5b includes the result for the masking percentage varying from 10 to 90. It can be observed that sparse coding-based recognition is indeed insensitive to occlusion to some degree: about 30% occlusion degrades the recognition performance by about 5%. This is not surprising, because the lower part of the human body is not as discriminating as the upper part (more theoretical justifications can be found in the paper [14]). Combined with the result in Figure 5a, we conclude that when spatial clue becomes less reliable (e.g., due to occlusion), it is plausible to exploit temporal ones by a strategy, such as tracking-for-recognition.

Finally, we use experimental results to clarify the importance of obtaining a good dictionary for sparse coding-based recognition. One basic assumption behind sparse coding-based recognition is that the dictionary contains a densely sampled representation of appearance subspace; such an assumption is not always valid in practical situations. For instance, if the training set and testing set are significantly different (e.g., without and with a bag), the accuracy of recognition will be affected. Table 1 includes the experimental results of sparse coding-based recognition on CASIA Dataset C for a variety of different training/testing set situations. It shows that the walking speed of the pedestrian has a minor impact on the recognition performance; while the effect of carrying a bag or not is substantial. This is in contrast to what we have observed for the tracking experiments, where handing a bag over does not affect the result much. Nevertheless, the recognition accuracy achieved by SCR (even in the situation of no fusion being involved) is at least comparable to the template-matching-based approach, as reported in [37]. One can expect that much better recognition performance can be obtained by temporal fusion, as we have shown above.

## 5. Conclusions

In this paper, we studied a unified approach toward robust pedestrian tracking and recognition from FLIR video via sparse coding. Under the joint tracking-and-recognition framework, tracking helps recognition by generating matching templates needed for the dictionary and by facilitating the exploitation of temporal redundancy; recognition helps multi-object recognition by supplying a nonlocal template updating strategy instead of a local one. The main contributions of this work include: (1) an automatic night biometrics system capable of tracking and recognizing pedestrians from infrared video; and (2) an extension of sparse coding-based tracking from a single target to multiple targets, enabled by the proposed recognition-based template updating strategy. We have reported our experimental results on two FLIR video data sets: the CASIA gait database and the WVU Infrared Pedestrian database. On the former, we show how joint tracking-and-recognition can improve the accuracy and robustness of sparse coding-based recognition; on the latter, we demonstrate that the nonlocal template updating strategy based on the recognition result is capable of boosting the performance of sparse coding-based tracking in the presence of occlusion.

## Figures and Tables

**Table 1.** The recognition performance of the baseline algorithm for the training/testing data of different conditions.

Training	Testing	This Work	[37]

Normal	Normal	91.05%	94%
Normal	Slow	84.05%	85%
Normal	Fast	88.35%	88%
Slow	Normal	81.24%	-
Fast	Normal	83.70%	-

with bag	with bag	93.56%	-
w/obag	w/o bag	92.94%	-
w/o bag	with bag	57.61%	51%
with bag	w/o bag	49.75%	-
